# Supplementary figures and images for: Impact of Time Point of Extracorporeal Membrane Oxygenation on Mortality and Morbidity in Congenital Diaphragmatic Hernia: A Single-Center Case Series
Source: Children (Basel). 2022 Jul 1;9(7):986. doi: 10.3390/children9070986 (PMC9315500; doi:10.3390/children9070986)

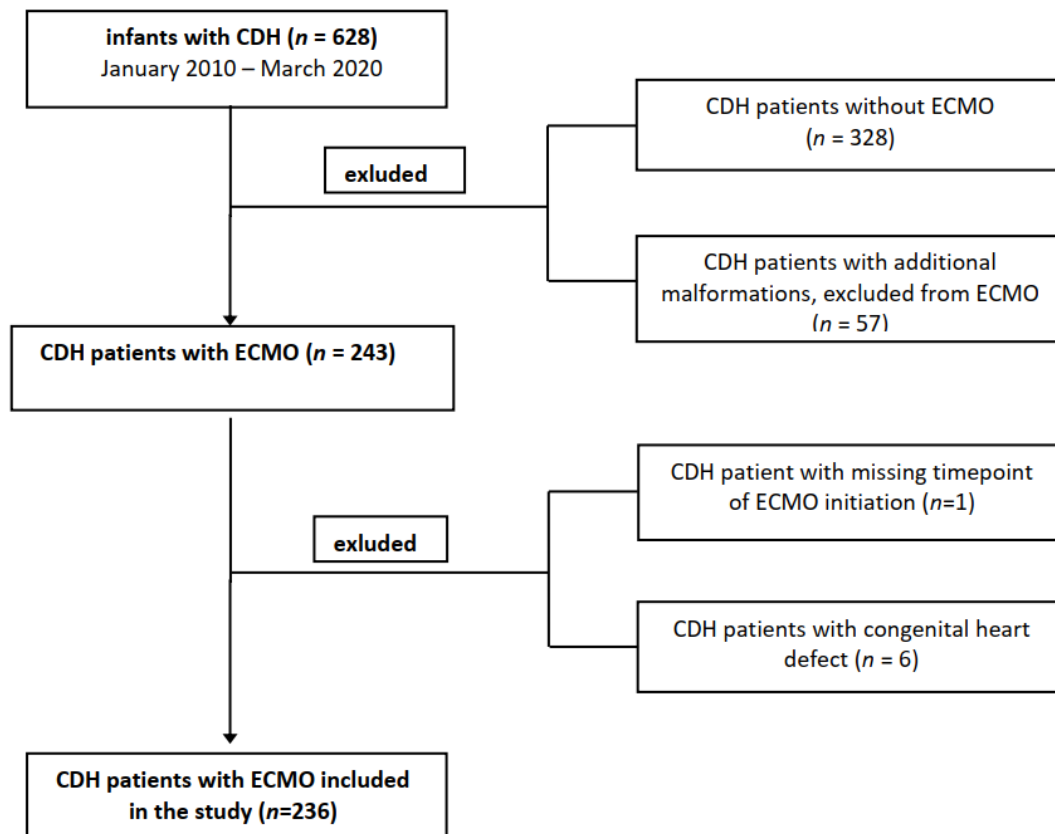

Figure S1. Recruitment of study population.

Supplement: Supplementary file 1 [file children-09-00986-s001.zip › children-1798476-supplementary.pdf]
